# Mendelian randomisation: A powerful and inexpensive method for identifying and excluding non-genetic risk factors for colorectal cancer

**DOI:** 10.1016/j.mam.2019.01.002

**Published:** 2019-10

**Authors:** Alex J. Cornish, Ian P.M. Tomlinson, Richard S. Houlston

**Affiliations:** a Division of Genetics and Epidemiology, The Institute of Cancer Research, London, UK; b Cancer Genetics and Evolution Laboratory, Institute of Cancer and Genomic Sciences, College of Medical and Dental Sciences, University of Birmingham, Birmingham, UK; c Department of Histopathology, University Hospitals Birmingham, Birmingham, UK; d Division of Molecular Pathology, The Institute of Cancer Research, London, UK

**Keywords:** Colorectal cancer, Mendelian randomisation, Risk factors, Causal relationship, Genetic variants, Instrumental variables

## Abstract

Colorectal cancer (CRC) is the third most common cancer in economically developed countries and a major cause of cancer-related mortality. The importance of lifestyle and diet as major determinants of CRC risk is suggested by differences in CRC incidence between countries and in migration studies. Previous observational epidemiological studies have identified associations between modifiable environmental risk factors and CRC, but these studies can be susceptible to reverse causation and confounding, and their results can therefore conflict. Mendelian randomisation (MR) analysis represents an approach complementary to conventional observational studies examining associations between exposures and disease. The MR strategy employs allelic variants as instrumental variables (IVs), which act as proxies for non-genetic exposures. These allelic variants are randomly assigned during meiosis and can therefore inform on life-long exposure, whilst not being subject to reverse causation. In previous studies MR frameworks have associated several modifiable factors with CRC risk, including adiposity, hyperlipidaemia, fatty acid profile and alcohol consumption. In this review we detail the use of MR to investigate and discover CRC risk factors, and its future applications.

## 1 Introduction

Colorectal cancer (CRC) is one of the most common cancers in economically developed countries and a major cause of cancer-related mortality (Forman et al., 2014). The disease is currently diagnosed in over one million individuals worldwide annually; although its incidence is set to rise in developing countries with the adoption of western lifestyles and diets (Haggar and Boushey, 2009). The importance of lifestyle and diet as major determinants of CRC risk have been strongly suggested by geographical differences in CRC incidence and demonstrated in migration studies (Kamangar et al., 2006). Given the importance of these factors in CRC risk, the modification of lifestyle and diet through public health initiatives offers the prospect of significant impact on CRC incidence. The full compendium of exposures affecting CRC risk, and their relative contributions, has yet to be elucidated however, necessitating further work to discover and validate risk factors.

## 2 Established and postulated colorectal cancer risk factors

Both environmental and genetic factors play important roles in CRC aetiology. The majority of CRCs are sporadic, with approximately 80% of patients presenting without a family history of the disease (Winawer et al., 1997). The lifetime risk for CRC in Western populations is approximately 4% (Siegel et al., 2017), although this risk is almost doubled in individuals with a first-degree family member diagnosed with CRC, and tripled in individuals with two or more affected family members (Taylor et al., 2010). Epidemiological studies have provided support for a hereditary component to the aetiology of a large number of cancers, including CRC (Risch, 2001). For many cancers, a higher concordance in monozygotic twins as compared with dizygotic twins, or with siblings, has been observed (Lichtenstein et al., 2000). Whilst such concordance is compatible with inherited genetic variation affecting risk, non-genetic mechanisms cannot be excluded as a basis of the measured heritability.

Technological developments in high-throughput genotyping and improved understanding of common genetic variation have made genome-wide association studies (GWAS) possible, facilitating the identification of single nucleotide polymorphisms (SNPs) influencing cancer risk (Sud et al., 2017). So far, GWAS have identified approximately 100 SNPs independently associated with CRC risk (Al-Tassan et al., 2015; Broderick et al., 2007; Cogent Study et al., 2008; Dunlop et al., 2012; Houlston et al., 2010; Kinnersley et al., 2012; Orlando et al., 2016; Schumacher et al., 2015; Tenesa et al., 2008; Tomlinson et al., 2007; Whiffin et al., 2014), with the largest study to-date combining data from over 125,000 individuals (Huyghe et al., 2019). The identification of these risk variants has contributed to an improved understanding of the pathways and mechanisms influencing CRC development.

Multiple lifestyle and environmental factors, many of which are modifiable, are now known to influence CRC risk (Kuipers et al., 2015; World Cancer Research Fund, 2017). The World Cancer Research Fund (2017) conducted a systematic analysis of known and suspected CRC risk factors, categorizing them into those with strong and limited evidence for a causal relationship. The evidence that obesity, height, alcohol intake and consumption of red and processed meats increases CRC risk was reported as strong, whilst the evidence that low intake of non-starchy vegetables and fruits, and high intake of foods containing iron increases CRC risk was reported to be weaker. The World Cancer Research Fund report also highlighted physical activity, the consumption of whole grains, fibre, dairy products and the use of calcium supplements as being strongly associated with lower CRC risk, but reported weaker evidence that use of multivitamin supplements, and high intake of foods containing vitamins C and D similarly decreases CRC risk.

Increased CRC risk has also been associated with chronic colitis due to inflammatory bowel disease (IBD) (Lu et al., 2018). The longer the duration of IBD, the greater the increase in CRC risk (Lu et al., 2018). However, whilst IBD is thought to increase risk of CRC, it explains only a small proportion of CRC incidence in Western populations (Kuipers et al., 2015). Improved surveillance and the increasing effectiveness of anti-inflammatory treatments may also be lowering the incidence of CRC in those with IBD (Jess et al., 2012). Heavy smoking over a prolonged period has also been recognised as being associated with increased CRC risk, in the region of an additional 11 cases per 100,000 person-years (Botteri et al., 2008).

The influence of modifiable environmental and lifestyle risk factors is likely to partly explain the socioeconomic and geographic differences in CRC rates (Doubeni et al., 2012). It has been estimated that as many as 71% of CRCs in Western countries may be due to these modifiable exposures and therefore preventable (Platz et al., 2000). However, to reduce CRC incidence through public health initiatives, it is imperative to determine which factors associated with CRC risk are causally related, and which are simply correlated.

## 3 Approaches for risk factor discovery and validation

### 3.1 Observational epidemiological studies

So far, many studies attempting to evaluate relationships between possible risk factors and diseases, such as CRC, have relied upon observational case-control, cohort, or cross-sectional study designs (Lawlor et al., 2004). Whilst observational studies have seemingly robustly associated a risk factor and a disease, interventions modifying such risk factors do not always result in the anticipated change in disease incidence (Davey Smith and Hemani, 2014). A number of explanations for these ostensibly paradoxical observations have been suggested, including the susceptibility of observational epidemiological studies to certain biases, such as reverse causation, confounding and errors in measurement. These biases can result in an apparent association between a risk factor and a disease, without the existence of a direct causal relationship.

Reverse causation occurs when the postulated risk factor does not itself influence disease development, but instead the occurrence of the disease affects the postulated risk factor. An example of possible reverse causation is in the relationship between computerized tomography (CT) scans and cancer (Mathews et al., 2013). Higher cancer incidence has been observed in individuals exposed to low-dose ionizing radiation from diagnostic CT scans (Mathews et al., 2013). However, it is difficult to exclude the possibility that the symptoms of precancerous conditions, or early cancer symptoms, led to patients having CT scans. Retrospective studies that seek to establish risk factors after disease diagnosis are especially susceptible to such reverse causation.

Confounders are factors causally related to both the postulated risk factor and the disease under consideration. For instance, smoking has been associated with increased risk of CRC (Botteri et al., 2008). However, smoking may be associated with other CRC risk factors, including alcohol consumption, physical inactivity and low uptake of CRC screening (Chao et al., 2000). An individual who consumes more alcohol is indeed more likely to smoke, and if alcohol consumption is causally related to CRC development, then this confounder could partly explain the association between smoking and CRC incidence. Such confounders are not always measured in observational epidemiological studies, and it may therefore not be possible to control for them when evaluating risk factors. Furthermore, unidentified confounders may exist, resulting in additional bias that could be impossible to account for in a traditional observational study design.

### 3.2 Mendelian randomisation

The gold standard for inferring causality is a randomized control trial (RCT). Individuals in an RCT are randomly assigned to two or more groups, minimizing both selection bias and confounding. As groups are assigned at the start of the study, reverse causation can be avoided. RCTs are however often not possible due to the costs or the time required. Furthermore, RCTs can have short follow-up times and hence only reflect the effect of an exposure at a certain time in life. It is also not always possible to assign individuals to groups to evaluate certain risk factors because of practical or ethical concerns.

Mendelian randomisation (MR) uses genetic variants, such as SNPs, as proxies for exposures to determine the effect of an exposure on an outcome (Sheehan et al., 2008). In the general population, germline variants tend to be randomly distributed with respect to most human traits. This occurs because of the fixed nature of germline genotypes and Mendel's laws of inheritance (*i.e.* segregation and independent assortment). Using germline variants as proxies for an exposure therefore ensures that MR is less susceptible to biases that affect many observational epidemiological studies (Davey Smith and Hemani, 2014). For example, as genotypes are randomly assigned at conception, MR is not biased by reverse causation. MR can therefore be considered analogous to a natural RCT. Furthermore, as genotypes are present at conception, MR analyses can examine the lifetime effect of an exposure on disease risk, unlike other study designs.

**Fig. 1:**
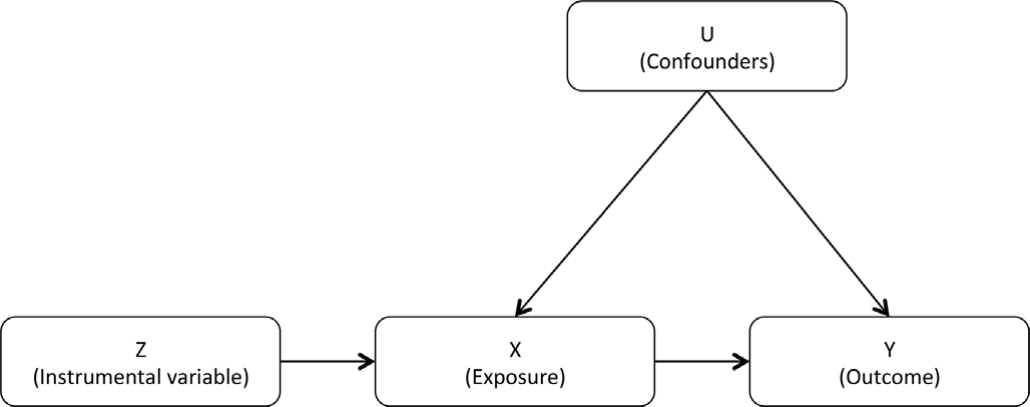
**The basic instrumental variable (IV) model depicted using a directed acyclic graph**. Z: the instrumental variable, X: the exposure of interest (such as a putative risk factor), Y: the outcome of interest (such as a disease), U: one or more measured or unmeasured confounders.

MR analyses involve three main assumptions: (i) the genetic variants used as IVs are robustly associated with the exposure under consideration; (ii) these IVs are independent of confounding factors; and (iii) the IVs are only associated with the outcome under consideration via the exposure (Lawlor et al., 2008). These three assumptions are often depicted as a directed acyclic graph (Fig. 1). The satisfaction of these three assumptions is sufficient to test the null hypothesis that the exposure is not causally related to the outcome. However, to accurately estimate the size of the effect, a fourth additional assumption is required: (iv) all associations depicted in Fig. 1 are unaffected by statistical interactions and are linear (Lawlor et al., 2008). Considering these assumptions, genetic variants can be used as proxies for a large range of modifiable risk factors. In one-sample MR, a single data set containing all information on the genetic variants, the exposure and the outcome for all individuals is used to assess a potential causal relationship (Haycock et al., 2016). In practice, few data sets contain exposure data for all individuals and a sufficient number of disease cases and controls to conduct one-sample MR analyses with sufficient power to identify causal effects. This has prompted the development of two-sample MR strategies, which use data from separate data sets: one containing information on genetic variants and the exposure of interest, and another containing information on the same genetic variants and the considered outcome (Hartwig et al., 2016). For many risk factors and cancers, GWAS containing data from tens or hundreds of thousands of individuals have been completed (Sud et al., 2017), facilitating the use of two-sample MR to study the causal relationship between various exposures and diseases. Resources such as the GWAS Catalog have collated and standardized data from a large number of studies (Buniello et al., 2019), aiding the identification of suitable genetic instruments.

One of the central tenets of MR is the absence of pleiotropy (*i.e.* a variant influencing multiple traits) between the SNPs associated with the exposure and outcome (Davey Smith and Hemani, 2014). Directional pleiotropy can result in the false identification of an association between a putative exposure and outcome, or the failure to identify a true causal relationship (Burgess and Thompson, 2017). Multiple methods have been developed to assess whether such directional pleiotropy exists and avoid it biasing causal estimates. In MR-Egger regression, the slope coefficient from Egger regression is used to assess directional pleiotropy (Bowden et al., 2015). MR-Egger regression relies on the assumption that the pleiotropic effects of each variant are independently distributed from the genetic association with the outcome; an assumption referred to as InSIDE (INstrument Strength Independent of Direct Effect). If the InSIDE assumption is satisfied, then MR-Egger causal effect estimates will be consistent as sample size and the number of genetic variants increase (Burgess and Thompson, 2017). Furthermore, under the InSIDE assumption the MR-Egger intercept term can be used to evaluate the average pleiotropic effect across variants. If this average pleiotropic effect is zero, then the MR-Egger causal effect estimate will equal causal effect estimates from other regression-based MR approaches, including the inverse variance weighted (IVW) method (Burgess and Thompson, 2017).

Approaches such as the HEIDI outlier test, implemented as part of the Generalised Summary-data-based Mendelian Randomisation (GSMR) package (Zhu et al., 2018), can also be used to identify and remove variants that violate IV assumptions. Methods such as simple and weighted median estimators (Bowden et al., 2016) and the mode-based estimate (Hartwig et al., 2017) can produce unbiased estimates of a causal effect even when the majority of IVs are invalid. Further methodological developments, such as latent causal variable (LCV) models, allow for accurate inference of causal relationships, even when the exposure and outcome are genetically correlated (O'Connor and Price, 2018). These methods also have their disadvantages however, with MR-Egger, median estimators and mode-based estimates generally achieving less power than conventional IV approaches (Bowden et al., 2015), and LCV models requiring genome-wide summary statistics for both the exposure and outcome – data that are not always available.

## 4 Limitations of Mendelian randomisation

Whilst MR analyses complement observational epidemiological studies, and are less influenced by some biases, there are a number of limitations to the approach. MR methods rely on the availability of genetic instruments robustly associated with the exposure of interest that can be used as proxies. For some exposures, such as obesity and height, GWAS containing data from hundreds of thousands of individuals have been completed, resulting in the identification of hundreds of exposure-associated SNPs that explain a high proportion of the genetically associated exposure (Yengo et al., 2018). For other exposures no or few associated SNPs have been identified, thereby prohibiting their study using MR frameworks. If exposure-associated SNPs have been identified, but these SNPs explain only a small proportion of exposure variation, then MR analyses may not be sufficiently powered to detect causal effects, especially if the true effect sizes are weak (Smith and Ebrahim, 2004).

The study of some risk factors requires additional information not always available, even when using a two-sample MR framework. For example, different SNPs are associated with smoking initiation and number of cigarettes smoked per day (Tobacco and Genetics Consortium, 2010), and it is therefore necessary to stratify disease cases and controls by whether they have ever smoked to accurately estimate the effect size of smoking on disease risk. In many cancer GWAS, smoking status is not measured, prohibiting the accurate assessment of effect size.

Although implemented in a number of studies (Ahmad et al., 2015; Gage et al., 2017), conventional IV approaches in a two-sample setting may not accurately estimate the effect size of a binary exposure, such as smoking initiation, on a binary outcome, such as disease diagnosis. Through simulation we previously evaluated whether IVW, weighted median estimators, mode-base estimates and MR-Egger methods provide reliable estimates of causal effects when considering binary exposures and binary outcomes (Disney-Hogg et al., 2018). When a causal relationship was simulated, the magnitudes of the effect estimates were inflated, and two-sample MR frameworks may therefore not be suitable for assessing the effect size of binary exposures on CRC risk.

## 5 Mendelian randomisation in colorectal cancer research

Many studies have implemented two-sample MR frameworks to evaluate causal relationships between risk factors and CRC. Here we discuss findings and insights from these studies (Table 1).

**Table 1:** MR studies that have assessed a possible causal relationship between exposures and CRC risk.

Risk factor	Reference	Number of CRC cases	Number of controls	Primary findings using MR
Adiponectin	Song et al. (2015)	7020	7631	No significant association.
	Nimptsch et al. (2017)	1253	1627	No significant association.
	Pei et al. (2015)	NA	NA	Inconsistent evidence.
Age at menarche & menopause	Neumeyer et al. (2018)	12,944	10,741	No significant association.
Alcohol	Wang et al. (2011)	2392	3951	Alcohol consumption associated with increased CRC risk (OR: 1.31, 95% CI: 1.01–1.70).
Aspirin	Lin et al. (2002)	161	219	No significant association.
C-reactive protein (CRP)	Wang et al. (2018)	30,480	22,844	No significant association.
	Nimptsch et al. (2015a)	727	727	CRP concentration associated with increased CRC risk (OR: 1.74, 95% CI: 1.06–2.85).
Fatty acids	May-Wilson et al. (2017)	9254	18,386	Arachidonic acid (OR: 1.05, 95% CI: 1.02–1.07, *P* = 1.7 × 10^−4^) and stearic saturated fatty acids (OR: 1.17, 95% CI: 1.01–1.35, *P* = 0.041) associated with increased CRC risk. Oleic (OR: 0.77, 95% CI: 0.65–0.92, *P* = 3.9 × 10^−3^) and palmitoleic (OR: 0.36, 95% CI: 0.15–0.84, *P* = 0.018) monounsaturated fatty acids, and linoleic polyunsaturated fatty acids (OR: 0.95, 95% CI: 0.93–0.98, *P* = 3.7 × 10^−4^) associated with reduced CRC risk.
Fetuin-A	Nimptsch et al. (2015b)	456	456	No significant association.
Height	Thrift et al. (2015a)	10,226	10,286	Height associated with increased CRC risk (OR: 1.07, 95% CI: 1.01–1.14).
	Khankari et al. (2016)	5100	4831	Height associated with increased CRC risk (OR: 1.58, 95% CI: 1.14–2.18, *P* = 0.006).
Interleukin-6	Tian et al. (2015)	10,257	12,391	No significant association.
Lipids	Rodriguez-Broadbent et al. (2017)	9254	18,386	Higher total cholesterol associated with increased CRC risk (OR: 1.46, 95% CI: 1.20–1.79, *P* = 1.68 × 10^−4^). Statin-effect-simulating *HMGCR* polymorphism associated with reduced CRC risk (OR: 0.69, 95% CI: 0.49–0.99, *P* = 0.046). No significant associations for low-density lipoprotein, high-density lipoprotein and total triglyceride.
Obesity	Jarvis et al. (2016)	9254	18,386	BMI (OR: 1.23, 95% CI: 1.02–1.49, *P* = 0.033), childhood obesity (OR: 1.07, 95% CI: 1.03–1.13, *P* = 0.018) and WHR (OR: 1.59, 95% CI: 1.08–2.34, *P* = 0.019) associated with increased CRC risk.
	Thrift et al. (2015b)	10,226	10,286	BMI associated with increased CRC risk (OR: 1.50, 95% CI: 1.13–2.01).
	Gao et al. (2016)	5100	4831	Adult BMI (OR: 1.39, 95% CI: 1.06–1.82, *P* = 0.016) associated with increased CRC risk. Childhood BMI and WHR non-significant.
Telomere length	Telomeres Mendelian Randomization Collaboration et al. (2017)	14,537	16,922	No significant association.
	Zhang et al. (2015)	5100	4831	No significant association.
Vitamin D	Dimitrakopoulou et al. (2017)	11,488	84,418	No significant association.
	He et al. (2018)	10,725	30,794	No significant association.
	Theodoratou et al. (2012)	2001	2237	No significant association.

Several studies have investigated the causal relationship between obesity-related factors and CRC risk. In a study of 9254 CRC cases and 18,386 controls, Jarvis et al. (2016) associated body mass index (BMI) (OR: 1.23, 95% CI: 1.02–1.49, *P* = 0.033), childhood obesity (OR: 1.07, 95% CI: 1.03–1.13, *P* = 0.018) and waist-hip ratio (WHR) (OR: 1.59, 95% CI: 1.08–2.34, *P* = 0.019) with increased CRC risk. Gao et al. (2016) similarly associated adult BMI with increased CRC risk (OR: 1.39, 95% CI: 1.06–1.82, *P* = 0.016), but did not find significant associations for childhood BMI or WHR, possibly due to the smaller size of their study, which contained 5100 CRC cases and 4831 controls. Many obesity-related traits have strong genetic correlations (Bulik-Sullivan et al., 2015) and further work is therefore necessary to investigate which aspects of adiposity have the greatest influence on CRC risk.

It has been suggested that adiponectin, an adipocyte-derived peptide hormone, may mediate the association between obesity and risk of CRC (Vansaun, 2013). Observational epidemiological studies of adiponectin have however yielded inconsistent results, with some associating lower circulating adiponectin with increased CRC risk (Aleksandrova et al., 2012), and others failing to identify such association (Stocks et al., 2008). MR analyses of adiponectin and CRC risk have also been inconsistent. In a multi-ethnic meta-analysis, Pei et al. (2015) considered five *ADIPOQ* polymorphisms, finding the genotype of one (rs2241766) to be associated with CRC risk (OR: 1.26, 95% CI: 1.09–1.47, *P = *0.002). Nimptsch et al. (2017) created an *ADIPOQ* allele score explaining approximately 3% of the variation in circulating adiponectin, but did not find this to be significantly associated with CRC risk. Song et al. (2015) considered 19 adiponectin-associated SNPs and similarly did not find any to be significantly associated with the risk of CRC. Inconsistencies between the observational epidemiological and MR studies could be due to reverse causation or confounding factors biasing the observational studies, or because of the relatively low power of the MR analyses to identify a causal relationship.

Hyperinsulinemia has also been suggested to be associated with increased risk of CRC. Nimptsch et al. (2015b) therefore used an MR framework to investigate whether fetuin-A, a liver protein known to inhibit the action of insulin, was causally related to CRC risk. No significant association was identified, although the analysis contained only 456 case-control pairs, and a small effect of fetuin-A on CRC risk therefore cannot be excluded.

Development of CRC has been positively correlated with circulating levels of plasma cholesterol and other components of the lipid profile in prospective epidemiological studies (Yao and Tian, 2015). It is not clear however whether these findings reflect a causal relationship or are the consequence of confounding by factors such as a common aetiology of both hyperlipidaemia and CRC. Lipid levels can be modified by both treatment with statins and lifestyle changes and an understanding of the causal relationship with CRC is therefore important when developing CRC prevention programs. The effect of statins, which reduce circulating cholesterol levels, on CRC risk is highly controversial, with a recent meta-analysis of eight RCTs failing to identify a significant beneficial effect (Lytras et al., 2014). Other analyses have however associated statin usage with lower CRC incidence (Mamtani et al., 2016). Rodriguez-Broadbent et al. (2017) employed MR frameworks to study the effects of blood levels of total cholesterol (TC), triglycerides (TG), low-density lipoprotein (LDL) and high-density lipoprotein (HDL) on CRC risk. Higher concentrations of TC were associated with an increased risk of CRC (OR: 1.46, 95% CI: 1.20–1.79, *P* = 1.68 × 10^−4^). Furthermore, a genetic risk score for *HMGCR*, simulating the effect of statins, was associated with reduced CRC risk (OR: 0.69, 95% CI: 0.49–0.99, *P* = 0.046) (Rodriguez-Broadbent et al., 2017). This study therefore supports a causal relationship between TC and CRC risk, providing further evidence that statin use could be effective in public health strategies aiming to reduce CRC incidence.

Dietary fat has been implicated as a cancer risk factor, with meta-analyses of epidemiological studies tending to associate higher consumption of red and processed meat with increased CRC risk (Aykan, 2015). It is unlikely however that the relationship between fat intake and CRC risk depends solely on the quantity, but also on the specific fatty acid (FA) type. Epidemiological studies and animal models have implicated animal fat (Reddy, 2002), some omega-6 polyunsaturated fatty acids (PUFAs) and saturated fatty acid (SFA) with increased cancer risk, and omega-3 PUFA consumption with reduced cancer risk (Azrad et al., 2013). The evidence of a causal relationship between the consumption of specific fatty acids from observational epidemiological studies has however been inconclusive. Possible reasons for this include reverse causation, confounding factors and inaccurate measurement of long-term diet (Theodoratou et al., 2007). Results from an MR study by May-Wilson et al. (2017) were broadly consistent with a pro-inflammatory FA profile having a detrimental effect on risk of CRC. Arachidonic acid (OR: 1.05, 95% CI: 1.02–1.07, *P* = 1.7 × 10^−4^) and stearic saturated FAs (OR: 1.17, 95% CI: 1.01–1.35, *P* = 0.041) were associated with increased CRC risk, whilst oleic (OR: 0.77, 95% CI: 0.65–0.92, *P* = 3.9 × 10^−3^) and palmitoleic (OR: 0.36, 95% CI: 0.15–0.84, *P* = 0.018) monounsaturated FAs, and linoleic polyunsaturated FAs (OR: 0.95, 95% CI: 0.93–0.98, *P* = 3.7 × 10^−4^) were associated with reduced CRC risk. In the analysis by May-Wilson et al. (2017), the same SNP (rs102275), or a correlated SNP (rs174547), was used to infer causal relationships between multiple FAs and risk of CRC. These SNPs were used assuming that the exposure individually accounts for the effect on CRC, and the effect of the genetic variant on CRC risk is therefore counted twice, in that it is assigned to multiple FA exposures (Holmes et al., 2017). Consequently, such single locus MR analyses are unable to determine which FA primarily drives the relationship between FA profile and CRC risk.

Chronic inflammation has been identified as a risk factor for CRC (Grivennikov, 2013). Higher concentrations of C-reactive protein (CRP), a marker of inflammation, have been associated with an increased risk of CRC in observational epidemiological studies (Tsilidis et al., 2008). Considering only observational studies it is unclear however whether this relationship is causal, or a result of confounding factors. Wang et al. (2018) conducted the largest MR analysis of CRP concentration and CRC risk to-date, and failed to find evidence that CRP concentrations are causally related to risk of CRC. Nimptsch et al. (2015a) conversely found a positive relationship between CRP concentrations and CRC risk (OR: 1.74, 95% CI: 1.06–2.85). However, the study by Nimptsch et al. (2015a) considered only 727 CRC cases and 727 controls, whilst Wang et al. (2018) used data from 30,480 CRC cases and 22,844 controls. This suggests that the causal relationship reported by Nimptsch et al. (2015a) may be a false positive.

Numerous studies have associated height with increased risk of cancers, including breast, CRC, leukemia, non-Hodgkin lymphoma and malignant melanoma (Green et al., 2011). GWAS of height encompassing more than half a million individuals have been conducted, identifying SNPs that explain a substantial proportion of height variation (Yengo et al., 2018), and MR frameworks are therefore well suited to investigate the causal relationship between height and CRC. Using data from 10,226 CRC cases and 10,286 controls, Thrift et al. (2015a) found a positive association between height and CRC risk (OR: 1.07, 95% CI: 1.01–1.14). In a smaller study of 5100 cases and 4831 controls, Khankari et al. (2016) also reported a positive association (OR: 1.58, 95% CI: 1.14–2.18, *P* = 0.006).

Epidemiological studies of the effect of reproductive factors on risk of CRC have not been consistent (Martinez et al., 1997; Tsilidis et al., 2010). Neumeyer et al. (2018) employed MR frameworks to study the effect of age at menarche and age at menopause on CRC risk, using data from 12,944 women diagnosed with CRC, and 10,741 women without CRC, identifying no significant associations. The SNPs used as IVs for these reproductive factors explain only a small proportion of their variance however, and therefore although this study used data from a large number of CRC cases and controls, it had limited power to detect weak causal effects. Nevertheless, these results suggest that it is unlikely that age at menarche and menopause have substantial causal effects on CRC risk.

Polymorphisms altering the conversion rates of alcohol-metabolizing enzymes, leading to a build-up of excess acetaldehyde and thereby reducing heavy alcohol use, are prevalent in certain Asian populations (Eng et al., 2007). These polymorphisms, such as Glu487Lys in *ALDH2*, therefore offer the opportunity to use MR frameworks to investigate the effect of alcohol consumption on various traits. Wang et al. (2011) conducted a meta-analysis of MR studies using the Glu487Lys polymorphism to examine the relationship between alcohol consumption and risk of CRC, finding higher genetically predicted alcohol intake to be associated with increased CRC risk (OR: 1.31, 95% CI: 1.01–1.70). The lower frequency of these enzyme-altering alleles in non-Asian populations limits the use of MR to study risk factors in other groups. Large projects such as UK Biobank, which has genotyped SNPs and collected data on alcohol consumption in approximately 500,000 individuals (Bycroft et al., 2018), have led to the identification of SNPs associated with alcohol intake common in other populations (Clarke et al., 2017). Additional alcohol-associated SNPs will facilitate the wider use of MR frameworks to study the effect of alcohol consumption on disease risk.

Dimitrakopoulou et al. (2017) used an MR strategy to investigate the relationship between vitamin D and the risk of seven cancers, including CRC, employing SNPs associated with circulating 25-hydroxyvitamin D (25-OHD) as IVs. The analysis found little evidence that vitamin D was associated with increased risk of any of the cancers. He et al. (2018) and Theodoratou et al. (2012) both also similarly found non-significant associations between circulating 25-OHD and CRC risk. These data do not support the results of observational epidemiological studies, which have found circulating 25-OHD to be associated with decreased CRC risk (Ma et al., 2011), suggesting that the observational studies could be biased by reverse causality or confounding factors.

Interleukin-6 (IL-6) is thought to influence the progression of several forms of cancer (Kumari et al., 2016). An MR analysis of circulating IL-6 concentrations and cancer risk was performed by Tian et al. (2015), who did not find evidence of a causal relationship. This is concordant with the results of observational epidemiological studies, which have also not identified IL-6 concentrations to be associated with CRC risk (Zhou et al., 2014).

One of the earliest studies to use an MR framework to investigate CRC risk factors was conducted by Lin et al. (2002), who used a polymorphism in *PTGS2* (Val511Ala) to simulate the effects of aspirin, and thereby study the relationship between aspirin use and CRC risk. Non-significant negative associations between the aspirin-simulating allele and CRC risk were reported, although the study sample sizes were small. The Val511Ala polymorphism is not common in some populations, including Chinese, Japanese and Caucasians, prohibiting its study in many large GWAS data sets based on these ethnicities (Lin et al., 2002).

The causal relevance of telomere length to various cancers has also been interrogated using MR frameworks. Whilst two MR studies found genetically predicted longer telomeres to be associated with increased risk of some cancers, including glioma and lung, no association with CRC risk was identified (Telomeres Mendelian Randomization Collaboration et al., 2017; Zhang et al., 2015). Retrospective observational studies of telomere length and CRC found individuals diagnosed with CRC to have shorter telomeres (Pooley et al., 2010), whilst a prospective study identified no significant association between telomere length and CRC diagnosis (Zee et al., 2009). The results from the prospective study and MR studies suggest that the inverse relationship identified in the retrospective study is likely due to reverse causation (Fernandez-Rozadilla et al., 2018; Pooley et al., 2010).

## 6 Future uses of Mendelian randomisation

Whilst MR has provided supporting evidence for a number of known and suspected CRC risk factors (Table 1), there are many other putative CRC risk factors that have not yet been interrogated using MR frameworks, including coffee consumption and intake of foods containing calcium, iron or zinc (World Cancer Research Fund, 2017). For some exposures, a lack of associated SNPs explaining a substantial proportion of variance prevents their consideration. As GWAS sample sizes continue to increase, the number of exposures for which there is sufficient power to identify small or moderate effect sizes under an MR framework will grow.

So far, MR analyses of CRC risk have generally been hypothesis-driven (*i.e.* have considered exposures for which there is pre-existing evidence for an effect on CRC risk). Hypothesis-free MR has the potential to identify previously unsuspected risk factors not considered in observational epidemiological studies (Evans and Davey Smith, 2015). Tools such as MR-Base (Hemani et al., 2018), which provides MR method implementations and databases of collated GWAS summary statistics, could help facilitate such hypothesis-free scans.

The development and application of additional techniques will also help robustly infer causality between exposures and CRC risk, whilst avoiding biases that can lead to false positives. GSMR improves upon the power of other summary-data-based MR methods by accounting for linkage disequilibrium between SNPs (Zhu et al., 2018), thereby avoiding the unnecessary loss of information. LCV models can identify causal relationships between genetically correlated traits, mediating such correlations with latent causal variables (O'Connor and Price, 2018). This reduces the number of false positives that can occur when using other summary-data-based MR approaches with genetically correlated traits (O'Connor and Price, 2018).

## 7 Conclusion

Mendelian randomisation has provided evidence supporting, and not supporting, the causal relationship between multiple risk factors and CRC. Further study using MR frameworks will help inform public health strategies, as well as provide better understanding of CRC aetiology.

## References

[bib1] Ahmad O.S. (2015). A Mendelian randomization study of the effect of type-2 diabetes on coronary heart disease. Nat. Commun..

[bib2] Al-Tassan N.A. (2015). A new GWAS and meta-analysis with 1000Genomes imputation identifies novel risk variants for colorectal cancer. Sci. Rep..

[bib3] Aleksandrova K. (2012). Total and high-molecular weight adiponectin and risk of colorectal cancer: the european prospective investigation into cancer and nutrition study. Carcinogenesis.

[bib4] Aykan N.F. (2015). Red meat and colorectal cancer. Onco Rev..

[bib5] Azrad M., Turgeon C., Demark-Wahnefried W. (2013). Current evidence linking polyunsaturated fatty acids with cancer risk and progression. Front Oncol.

[bib6] Botteri E. (2008). Smoking and colorectal cancer: a meta-analysis. J. Am. Med. Assoc..

[bib7] Bowden J., Davey Smith G., Burgess S. (2015). Mendelian randomization with invalid instruments: effect estimation and bias detection through Egger regression. Int. J. Epidemiol..

[bib8] Bowden J. (2016). Consistent estimation in mendelian randomization with some invalid instruments using a weighted median estimator. Genet. Epidemiol..

[bib9] Broderick P. (2007). A genome-wide association study shows that common alleles of SMAD7 influence colorectal cancer risk. Nat. Genet..

[bib10] Bulik-Sullivan B. (2015). An atlas of genetic correlations across human diseases and traits. Nat. Genet..

[bib99] Buniello (2019). The NHGRI-EBI GWAS Catalog of published genome-wide association studies, targeted arrays and summary statistics 2019. Nucleic Acids Res..

[bib11] Burgess S., Thompson S.G. (2017). Interpreting findings from Mendelian randomization using the MR-Egger method. Eur. J. Epidemiol..

[bib12] Bycroft C. (2018). The UK Biobank resource with deep phenotyping and genomic data. Nature.

[bib13] Chao A. (2000). Cigarette smoking and colorectal cancer mortality in the cancer prevention study II. J. Natl. Cancer Inst..

[bib14] Clarke T.K. (2017). Genome-wide association study of alcohol consumption and genetic overlap with other health-related traits in UK Biobank (N=112 117). Mol. Psychiatr..

[bib15] Cogent Study (2008). Meta-analysis of genome-wide association data identifies four new susceptibility loci for colorectal cancer. Nat. Genet..

[bib16] Davey Smith G., Hemani G. (2014). Mendelian randomization: genetic anchors for causal inference in epidemiological studies. Hum. Mol. Genet..

[bib17] Dimitrakopoulou V.I. (2017). Circulating vitamin D concentration and risk of seven cancers: mendelian randomisation study. BMJ.

[bib18] Disney-Hogg L. (2018). Impact of atopy on risk of glioma: a Mendelian randomisation study. BMC Med..

[bib19] Doubeni C.A. (2012). Contribution of behavioral risk factors and obesity to socioeconomic differences in colorectal cancer incidence. J. Natl. Cancer Inst..

[bib20] Dunlop M.G. (2012). Common variation near CDKN1A, POLD3 and SHROOM2 influences colorectal cancer risk. Nat. Genet..

[bib21] Eng M.Y., Luczak S.E., Wall T.L. (2007). ALDH2, ADH1B, and ADH1C genotypes in Asians: a literature review. Alcohol Res. Health.

[bib22] Evans D.M., Davey Smith G. (2015). Mendelian randomization: new applications in the coming age of hypothesis-free causality. Annu. Rev. Genom. Hum. Genet..

[bib23] Fernandez-Rozadilla C. (2018). Telomere length and genetics are independent colorectal tumour risk factors in an evaluation of biomarkers in normal bowel. Br. J. Canc..

[bib24] Forman D. (2014).

[bib25] Gage S.H. (2017). Assessing causality in associations between cannabis use and schizophrenia risk: a two-sample Mendelian randomization study. Psychol. Med..

[bib26] Gao C. (2016). Mendelian randomization study of adiposity-related traits and risk of breast, ovarian, prostate, lung and colorectal cancer. Int. J. Epidemiol..

[bib27] Green J. (2011). Height and cancer incidence in the Million Women Study: prospective cohort, and meta-analysis of prospective studies of height and total cancer risk. Lancet Oncol..

[bib28] Grivennikov S.I. (2013). Inflammation and colorectal cancer: colitis-associated neoplasia. Semin. Immunopathol..

[bib29] Haggar F.A., Boushey R.P. (2009). Colorectal cancer epidemiology: incidence, mortality, survival, and risk factors. Clin. Colon Rectal Surg..

[bib30] Hartwig F.P. (2016). Two-sample Mendelian randomization: avoiding the downsides of a powerful, widely applicable but potentially fallible technique. Int. J. Epidemiol..

[bib31] Hartwig F.P., Davey Smith G., Bowden J. (2017). Robust inference in summary data Mendelian randomization via the zero modal pleiotropy assumption. Int. J. Epidemiol..

[bib32] Haycock P.C. (2016). Best (but oft-forgotten) practices: the design, analysis, and interpretation of Mendelian randomization studies. Am. J. Clin. Nutr..

[bib33] He Y. (2018). Exploring causality in the association between circulating 25-hydroxyvitamin D and colorectal cancer risk: a large Mendelian randomisation study. BMC Med..

[bib34] Hemani G. (2018). The MR-Base platform supports systematic causal inference across the human phenome. Elife.

[bib35] Holmes M.V., Ala-Korpela M., Smith G.D. (2017). Mendelian randomization in cardiometabolic disease: challenges in evaluating causality. Nat. Rev. Cardiol..

[bib36] Houlston R.S. (2010). Meta-analysis of three genome-wide association studies identifies susceptibility loci for colorectal cancer at 1q41, 3q26.2, 12q13.13 and 20q13.33. Nat. Genet..

[bib37] Huyghe J.R. (2019). Discovery of common and rare genetic risk variants for colorectal cancer. Nat. Genet..

[bib38] Jarvis D. (2016). Mendelian randomisation analysis strongly implicates adiposity with risk of developing colorectal cancer. Br. J. Canc..

[bib39] Jess T. (2012). Decreasing risk of colorectal cancer in patients with inflammatory bowel disease over 30 years. Gastroenterology.

[bib40] Kamangar F., Dores G.M., Anderson W.F. (2006). Patterns of cancer incidence, mortality, and prevalence across five continents: defining priorities to reduce cancer disparities in different geographic regions of the world. J. Clin. Oncol..

[bib41] Khankari N.K. (2016). Association between adult height and risk of colorectal, lung, and prostate cancer: results from meta-analyses of prospective studies and mendelian randomization analyses. PLoS Med..

[bib42] Kinnersley B. (2012). The TERT variant rs2736100 is associated with colorectal cancer risk. Br. J. Canc..

[bib43] Kuipers E.J. (2015). Colorectal cancer. Nat Rev Dis Primers.

[bib44] Kumari N. (2016). Role of interleukin-6 in cancer progression and therapeutic resistance. Tumour Biol.

[bib45] Lawlor D.A. (2004). Observational versus randomised trial evidence. Lancet.

[bib46] Lawlor D.A. (2008). Mendelian randomization: using genes as instruments for making causal inferences in epidemiology. Stat. Med..

[bib47] Lichtenstein P. (2000). Environmental and heritable factors in the causation of cancer--analyses of cohorts of twins from Sweden, Denmark, and Finland. N. Engl. J. Med..

[bib48] Lin H.J. (2002). Prostaglandin H synthase 2 variant (Val511Ala) in African Americans may reduce the risk for colorectal neoplasia. Cancer Epidemiol. Biomark. Prev..

[bib49] Lu M.J. (2018). Systematic review with meta-analysis: thiopurines decrease the risk of colorectal neoplasia in patients with inflammatory bowel disease. Aliment. Pharmacol. Ther..

[bib50] Lytras T., Nikolopoulos G., Bonovas S. (2014). Statins and the risk of colorectal cancer: an updated systematic review and meta-analysis of 40 studies. World J. Gastroenterol..

[bib51] Ma Y. (2011). Association between vitamin D and risk of colorectal cancer: a systematic review of prospective studies. J. Clin. Oncol..

[bib52] Mamtani R. (2016). Disentangling the association between statins, cholesterol, and colorectal cancer: a nested case-control study. PLoS Med..

[bib53] Martinez M.E. (1997). A prospective study of reproductive factors, oral contraceptive use, and risk of colorectal cancer. Cancer Epidemiol. Biomark. Prev..

[bib54] Mathews J.D. (2013). Cancer risk in 680,000 people exposed to computed tomography scans in childhood or adolescence: data linkage study of 11 million Australians. BMJ.

[bib55] May-Wilson S. (2017). Pro-inflammatory fatty acid profile and colorectal cancer risk: a Mendelian randomisation analysis. Eur. J. Cancer.

[bib56] Neumeyer S. (2018). Mendelian randomisation study of age at menarche and age at menopause and the risk of colorectal cancer. Br. J. Canc..

[bib57] Nimptsch K. (2015). Association of CRP genetic variants with blood concentrations of C-reactive protein and colorectal cancer risk. Int. J. Canc..

[bib58] Nimptsch K. (2015). Plasma fetuin-A concentration, genetic variation in the AHSG gene and risk of colorectal cancer. Int. J. Canc..

[bib59] Nimptsch K. (2017). Genetic variation in the ADIPOQ gene, adiponectin concentrations and risk of colorectal cancer: a Mendelian Randomization analysis using data from three large cohort studies. Eur. J. Epidemiol..

[bib60] O'Connor L.J., Price A.L. (2018). Distinguishing genetic correlation from causation across 52 diseases and complex traits. Nat. Genet..

[bib61] Orlando G. (2016). Variation at 2q35 (PNKD and TMBIM1) influences colorectal cancer risk and identifies a pleiotropic effect with inflammatory bowel disease. Hum. Mol. Genet..

[bib62] Pei Y., Xu Y., Niu W. (2015). Causal relevance of circulating adiponectin with cancer: a meta-analysis implementing Mendelian randomization. Tumour Biol.

[bib63] Platz E.A. (2000). Proportion of colon cancer risk that might be preventable in a cohort of middle-aged US men. Cancer Causes Control.

[bib64] Pooley K.A. (2010). Telomere length in prospective and retrospective cancer case-control studies. Cancer Res..

[bib65] Reddy B.S. (2002). Types and amount of dietary fat and colon cancer risk: prevention by omega-3 fatty acid-rich diets. Environ. Health Prev. Med..

[bib66] Risch N. (2001). The genetic epidemiology of cancer: interpreting family and twin studies and their implications for molecular genetic approaches. Cancer Epidemiol. Biomark. Prev..

[bib67] Rodriguez-Broadbent H. (2017). Mendelian randomisation implicates hyperlipidaemia as a risk factor for colorectal cancer. Int. J. Canc..

[bib68] Schumacher F.R. (2015). Genome-wide association study of colorectal cancer identifies six new susceptibility loci. Nat. Commun..

[bib69] Sheehan N.A. (2008). Mendelian randomisation and causal inference in observational epidemiology. PLoS Med..

[bib70] Siegel R.L. (2017). Colorectal cancer statistics, 2017. Ca - Cancer J. Clin..

[bib71] Smith G.D., Ebrahim S. (2004). Mendelian randomization: prospects, potentials, and limitations. Int. J. Epidemiol..

[bib72] Song M. (2015). Genetic variants of adiponectin and risk of colorectal cancer. Int. J. Canc..

[bib73] Stocks T. (2008). Components of the metabolic syndrome and colorectal cancer risk; a prospective study. Int. J. Obes..

[bib74] Sud A., Kinnersley B., Houlston R.S. (2017). Genome-wide association studies of cancer: current insights and future perspectives. Nat. Rev. Canc..

[bib75] Taylor D.P. (2010). Population-based family history-specific risks for colorectal cancer: a constellation approach. Gastroenterology.

[bib76] Telomeres Mendelian Randomization Collaboration (2017). Association between telomere length and risk of cancer and non-neoplastic diseases: a mendelian randomization study. JAMA Oncol.

[bib77] Tenesa A. (2008). Genome-wide association scan identifies a colorectal cancer susceptibility locus on 11q23 and replicates risk loci at 8q24 and 18q21. Nat. Genet..

[bib78] Theodoratou E. (2007). Dietary fatty acids and colorectal cancer: a case-control study. Am. J. Epidemiol..

[bib79] Theodoratou E. (2012). Instrumental variable estimation of the causal effect of plasma 25-hydroxy-vitamin D on colorectal cancer risk: a mendelian randomization analysis. PLoS One.

[bib80] Thrift A.P. (2015). Mendelian randomization study of height and risk of colorectal cancer. Int. J. Epidemiol..

[bib81] Thrift A.P. (2015). Mendelian randomization study of body mass index and colorectal cancer risk. Cancer Epidemiol. Biomark. Prev..

[bib82] Tian G. (2015). Circulating interleukin-6 and cancer: a meta-analysis using Mendelian randomization. Sci. Rep..

[bib83] Tobacco and Genetics Consortium (2010). Genome-wide meta-analyses identify multiple loci associated with smoking behavior. Nat. Genet..

[bib84] Tomlinson I. (2007). A genome-wide association scan of tag SNPs identifies a susceptibility variant for colorectal cancer at 8q24.21. Nat. Genet..

[bib85] Tsilidis K.K. (2008). C-reactive protein and colorectal cancer risk: a systematic review of prospective studies. Int. J. Canc..

[bib86] Tsilidis K.K. (2010). Oral contraceptives, reproductive history and risk of colorectal cancer in the European Prospective Investigation into Cancer and Nutrition. Br. J. Canc..

[bib87] Vansaun M.N. (2013). Molecular pathways: adiponectin and leptin signaling in cancer. Clin. Canc. Res..

[bib88] Wang J. (2011). Alcohol ingestion and colorectal neoplasia: a meta-analysis based on a Mendelian randomization approach. Colorectal Dis..

[bib89] Wang X. (2018). Mendelian randomization analysis of C-reactive protein on colorectal cancer risk. Int. J. Epidemiol..

[bib90] Whiffin N. (2014). Identification of susceptibility loci for colorectal cancer in a genome-wide meta-analysis. Hum. Mol. Genet..

[bib91] Winawer S.J. (1997). Colorectal cancer screening: clinical guidelines and rationale. Gastroenterology.

[bib92] World Cancer Research Fund (2017). Diet, Nutrition, Physical Activity and Colorectal Cancer.

[bib93] Yao X., Tian Z. (2015). Dyslipidemia and colorectal cancer risk: a meta-analysis of prospective studies. Cancer Causes Control.

[bib94] Yengo L. (2018). Meta-analysis of genome-wide association studies for height and body mass index in approximately 700000 individuals of European ancestry. Hum. Mol. Genet..

[bib95] Zee R.Y. (2009). Mean telomere length and risk of incident colorectal carcinoma: a prospective, nested case-control approach. Cancer Epidemiol. Biomark. Prev..

[bib96] Zhang C. (2015). Genetic determinants of telomere length and risk of common cancers: a Mendelian randomization study. Hum. Mol. Genet..

[bib97] Zhou B. (2014). C-reactive protein, interleukin-6 and the risk of colorectal cancer: a meta-analysis. Cancer Causes Control.

[bib98] Zhu Z. (2018). Causal associations between risk factors and common diseases inferred from GWAS summary data. Nat. Commun..

